# Modelling and Validation of Synthesis of Poly Lactic Acid Using an Alternative Energy Source through a Continuous Reactive Extrusion Process

**DOI:** 10.3390/polym8040164

**Published:** 2016-04-22

**Authors:** Satya P. Dubey, Hrushikesh A. Abhyankar, Veronica Marchante, James L. Brighton, Kim Blackburn, Clive Temple, Björn Bergmann, Giang Trinh, Chantal David

**Affiliations:** 1 Advanced Vehicle Engineering Centre (AVEC), School of Aerospace, Transport and Manufacturing (SATM), Cranfield University, MK43 0AL Cranfield, UK; s.p.dubey@cranfield.ac.uk (S.P.D.); v.marchanterodriguez@cranfield.ac.uk (V.M.); j.l.brighton@cranfield.ac.uk (J.L.B.); k.blackburn@cranfield.ac.uk (K.B.); C.Temple@cranfield.ac.uk (C.T.); 2 Polymer Engineering, Fraunhofer.-ICT, Joseph-von-Fraunhofer-Straße 7, 76327 Pfinztal, Germany; Bjoern.Bergmann@ict.fraunhofer.de; 3 Sciences Computers Consultants (SCC), 10 Rue du Plateau des Glières, 42000 Saint-Étienne, France; gtrinh@scconsultants.com (G.T.); chdavid@scconsultants.com (C.D.)

**Keywords:** alternative energy, bio-degradable, reactive extrusion, metal catalyst, mathematical modelling, poly lactic acid (PLA), ring opening polymerization (ROP)

## Abstract

PLA is one of the most promising bio-compostable and bio-degradable thermoplastic polymers made from renewable sources. PLA is generally produced by ring opening polymerization (ROP) of lactide using the metallic/bimetallic catalyst (Sn, Zn, and Al) or other organic catalysts in a suitable solvent. In this work, reactive extrusion experiments using stannous octoate Sn(Oct)_2_ and tri-phenyl phosphine (PPh)_3_ were considered to perform ROP of lactide. Ultrasound energy source was used for activating and/or boosting the polymerization as an alternative energy (AE) source. Ludovic^®^ software, designed for simulation of the extrusion process, had to be modified in order to simulate the reactive extrusion of lactide and for the application of an AE source in an extruder. A mathematical model for the ROP of lactide reaction was developed to estimate the kinetics of the polymerization process. The isothermal curves generated through this model were then used by Ludovic software to simulate the “reactive” extrusion process of ROP of lactide. Results from the experiments and simulations were compared to validate the simulation methodology. It was observed that the application of an AE source boosts the polymerization of lactide monomers. However, it was also observed that the predicted residence time was shorter than the experimental one. There is potentially a case for reducing the residence time distribution (RTD) in Ludovic^®^ due to the ‘liquid’ monomer flow in the extruder. Although this change in parameters resulted in validation of the simulation, it was concluded that further research is needed to validate this assumption.

## 1. Introduction

The production of large quantities of polymer waste is one of the major challenges of the present era, especially as polymers represent 20%–30% of global waste [1,2]. As a result of massive consumption of polymer products, problems such as decomposition and recycling of polymers have emerged. As alternatives, bio-mass and/or bio-degradable polymers such as cellulose acetate (CA), poly-butylene succinate (PBS), poly-lactic acid (PLA), poly-urethane (PU) *etc.*, have attracted interest. Among these bio-plastics, poly-lactide or poly lactic acid (PLA) has been the focus of increasing interest, with levels of industrial production becoming much more significant [2,3,4,5,6,7,8,9]. 

PLA polymer cross-linked with poly vinyl acetate (PVAc) and hexadecanoic acid (HA) exhibits shape memory behavior. This can be obtained by water driven actuation, thermal effect and electrical effect [10,11] and has a wide range of applications in areas such as aerospace engineering, textiles, automobiles, and medicinal stitches, *etc.*

For decades, the conventional commercial method for synthesis of PLA has been ring-opening polymerization (ROP) based on metal (Sn, Al, Zn) catalysis of lactide, using suitable co-catalyst such as dodecanol and solvent [1,12,13,14,15,16]. However, PLA production according to conventional methods can still lead to potential toxicity issues, linked to residual metal traces from the catalyst, which can limit the potential scope of applications. 

Attempts have been made to substitute tin-based catalysts by organic catalysts that exhibit less toxicity or eco-toxicity [17,18,19]. It has also been observed through initial experimental trials that alternative energy sources (LASER, Ultrasounds, microwaves) with the combination of a prominent catalyst source, could be an option to facilitate the ROP of lactide monomers [20,21,22,23,24,25,26,27]. It is a well-known fact that the efficiency of the metal catalyst, in general, must be very high in order to perform the polymerization; on the other hand, by replacing a metal catalyst with a metal-free catalyst, it is not easy to obtain a similar process efficiency. The implementation of a metal-free catalyst and the suitable application of an alternative energy source in the ROP of the lactide process may result in the replacement of the metal catalysts completely, thus achieving non-toxic PLA production at industrially acceptable rates (30–40) kg/h.

There are several projects underway with research groups trying to optimize the polymerization process of PLA through cleaner, more efficient and safer processes. One such project is InnoREX [20]. InnoREX aims to combine organo-metallic catalysis with reactive extrusion (REX) for ROP of lactide to obtain a high throughput. The project also aims to explore the use of alternative energy sources (microwave, ultrasound) to reduce the total energy consumption and improve eco-friendly design. To the best of the authors’ knowledge, there is a dearth of literature in the area of ROP of lactide through the use of ultrasound energy. The use of an ultrasound source was first reported by Jevtić *et al.* [27] as a thermal energy source for degradation of the PLA chain. An intense and targeted ultrasound source to separate the PLA chain from poly lactide-*co*-glycolic acid was one of the first attempts to use an AE source for polymer chain separation. This research led to the investigation of an ultrasound as an AE source for the ROP process, as it can provide considerable activation energy to initiate polymerization reaction [19,26,28].

**Figure 1:**
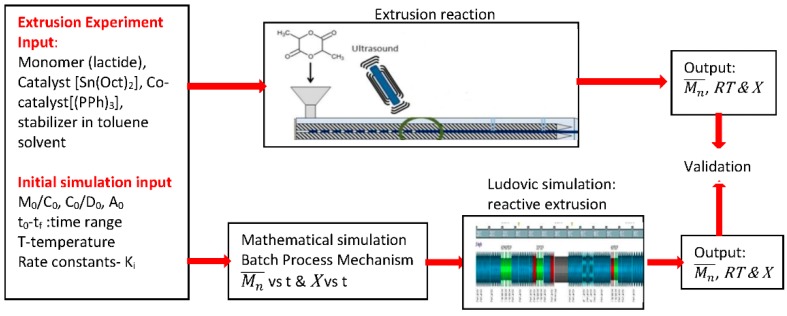
Methodology to explain reaction parameters. *M*_0_: Initial concentration of monomer, *C*_0_: Initial concentration of catalyst Sn(Oct)_2_, *D*_0_: Initial concentration of OH group source, *A*_0_: Initial concentration of phosphine (PPh)_3_, $\bar{(M_{n}}$) number average molecular weight, *X*: conversion, RT: residential time, *K*i: rate constants.

In this work, the impact of AE on ROP of PLA using a metal catalyst has been investigated through extrusion experiments and validated using Ludovic^®^ simulations. For this, the work has been divided into three different stages Figure 1: (1) Extrusion—to obtain experimental data of ROP of PLA using Sn(Oct)_2_ as catalyst and the application of ultrasound; (2) Simulation batch process—the model developed in previous work [29] was used to obtain isothermal curves; and (3) implementation of the isothermal curves (output of Step 2) in Ludovic^®^ software [30] to simulate reactive extrusion of ROP of PLA. The premise for using an AE source in the metal catalyst process was to reduce the role of the metal catalyst by supporting the AE source by enhancing the efficiency of the process. 

Experimental data for reactive extrusion of ROP of Lactide was obtained at the laboratory level. The experiments were conducted using stannous octoate as a catalyst. In addition, ultrasound source was applied to facilitate the mixing of monomer and catalyst.

On the other hand, the mathematical model to describe the polymerization of PLA in a batch process was presented in a previous work [29]. The reaction mechanism includes three basic stages in ROP reactions (initiation, propagation and termination) and other side reactions (trans-esterification, chain transfer, non-radical chain scission, *etc.*).

This model was implemented in Ludovic^®^. This was conducted using isothermal curves, with particular reference to the variation of number average molecular weight ($\bar{M_{n}}$) and conversion (*X*) with time. Ludovic^®^ uses the isothermal curves (provided as an input table) to estimate the stage and/or progress of the polymerization during the reactive extrusion. To the best of the authors’ knowledge, this is a significant step forward in the state-of-the-art in this area of research. In addition, the effect of AE has also examined in Ludovic^®^. Consideration of the AE source in large scale simulations through Ludovic^®^ software is another innovation in this area.

Finally, analysis and comparison of experimental data and simulated results are presented in this work. The results obtained through the reactive extrusion experiment were used to validate the output from the Ludovic^®^ simulation. This work also attempts to explain the discrepancies in the output from the software *vis-à-vis* reactive extrusion.

## 2. Materials and Processes

### 2.1. Materials Details

The monomer used for the reactive extrusion was l-lactide (Sigma-Aldrich, St. Louis, MO, USA), the catalyst, co-catalyst and initiator were stannous octoate [Sn(Oct)_2_] (Sn-bis-ethyl-2-hexanoate, Sigma-Aldrich), triphenylphosphine (TPP) (Sigma-Aldrich), and 1-dodecanol (Sigma-Aldrich), respectively. Toluene (Sigma-Aldrich) was used as a solvent.

### 2.2. Experimental Process: Reactive Extrusion of PLA

Reactive extrusion experiments were performed at the Fraunhofer, ICT laboratory [31], using Leistritz 27 HP (Leistritz Extrusionstechnik, Nürnberg, Germany), a co-rotating twin screw extruder. The configuration of the twin screw extruder used was: barrel dimensions 27 mm diameter and *L*/*D* = 52, temperature range (130–220 °C) ultrasound source, as an alternative source of energy applied in this process using a sonotrode “Ultrasound Sonification Device” UIP2000hdT (2000 W) (Hielscher Ultrasonic GmbH, Teltow, Germany) for the time range (3–15) min. The ultrasound power range used for mixing the material in the reaction process is (400–600) Watt. An extruder block was specifically designed to facilitate the application of ultrasound. In this block, the sonotrode was mounted and it was placed at a distance of 25 D from the feed for the reaction, while the monomer and catalysts were pre-mixed under an inert gas atmosphere and fed into the main feed. The initial concentration of the reactants and some initial conditions of the experiments are shown in Table 1. 

### 2.3. Characterisation Techniques 

Monomer conversion (*X*) and average molecular weight ($\bar{M_{n}}$) were measured at several temperatures, time and molecular monomer to initiator ratios. Samples were then taken at different stages of the reaction process to evaluate the polymerization efficiency. Details of the methods and devices used for analysis are listed below.

#### 2.3.1. Gel Permeation Chromatography (GPC)

Hexafluoroisopropanol (HFIP) was used as a solvent in GPC for the analysis of PLA polymer. GPC is equipped with a detector and differential refractive index. Depending on the molecular weight of the particular sample, a column technique was used with the calibration standard based on the Poly(methyl methacrylate) (PMMA) system. Details of the GPC configuration are as follows:

- Poly(styrene sulfonate) (PSS) (series 1100, Agilent, Missouri, United States), at 35 °C,
- Columns: [PSS PFG 7 µm × 8 mm × 50 mm (guard column) PSS PFG 7 µm × 8 mm × 300 mm; 100 A PSS PFG 7 µm × 8 mm × 300 mm; 1000 A].
- Flow: 1, 0 mL/min, Detector: Refractive Index Detector Agilent 1100.
- Injection volume: 100 µL.

This technique was used to determine the average molecular weight ($\bar{M_{n}}$) values. The values obtained through GPC are not an absolute measure but at the equilibrium.

#### 2.3.2. Proton Nuclear Magnetic Resonance (^1^H NMR)

Proton nuclear magnetic resonance analysis was conducted in CDCl_3_ using a Bruker (250) MHz spectrometer (Coventry, UK) at 30 °C in deuterated chloroform. The solution concentration used was 5 wt/vol %. This technique was applied to determine the monomer concentration and from that the conversion rate (*X)*. This analysis was also applied to the sample extracted from the extruder, which was a mixture of monomer and polymer. Several numbers of scans (100) were performed for each sample until the complete separation of polymer and monomers was achieved. 

## 3. Mathematical Modelling of Reactive Extrusion 

In general, the ROP of lactide is a complex process which involves several reaction stages. To assess the impact of the reaction parameters (concentration of monomer, the initial concentration of catalyst, the ratio among reactants, kinetic rate constants, AE source, temperature, *etc.*) on the efficiency of the polymerization (mainly conversion and average molecular weight), it is necessary to formulate a suitable reaction mechanism. Mathematical modelling is a powerful and cost-effective tool to analyze these parameters. It can also help to explain the thermodynamic behavior of the process and explains how it can be scaled up to commercial levels of production.

Developing a simulation model which defines the extrusion process (accounting for the polymerization reaction) is complex, due to the interdependencies of the reactions. This process was divided into two parts: firstly, the development of a mathematical model describing the ROP in a batch process and, secondly, integrating this model into Ludovic^®^ software (Saint-Etienne, France). From the ROP model for the batch process, isothermal curves for the evolution of number average molecular weight ($\bar{M_{n}}$) and conversion (*X*) with time were obtained. These curves were discretized and integrated into Ludovic^®^. This way, Ludovic^®^ was able to estimate a degree of conversion and growth of the polymer chains during the simulation of the reactive extrusion process. 

Reaction mechanisms can be defined for any ROP reaction (mechanisms will tend to be similar to the one we have defined). However, depending on the monomer, initiator, and catalyst and, indeed, the ratios of the monomer to catalyst and catalyst to the initiator, the reaction parameters will change, and as such they will have to always be validated against experiments. However, once the validation is undertaken, these can be used to model the process based on similar reaction conditions.

### 3.1. Reaction Kinetics Modelling and Mathematical Simulation of ROP of Lactide

In previous related work, Dubey *et al.* [29] covered the details of mathematical modelling of polymerization of PLA based on a five-stage reaction mechanism. The reaction mechanism comprises initiation, propagation, termination, trans-esterification and, probably, non-radical random chain scission. For continuity, the mechanism used is reproduced in Equations (1)–(9).

Activation of catalyst:

(1) $$C+D_{i}\begin{array}{c} \overset{k_{a1}}{\to }\\ \underset{k_{a2}}{\leftarrow } \end{array}R_{i}+A$$

Propagation:

(2) $$R_{i}+M\begin{array}{c} \overset{\;k_{p}}{\to }\\ \underset{\;k_{d}}{\leftarrow } \end{array}R_{i+1}$$

Chain Transfer:

(3) $$R_{i}+D_{j}\begin{array}{c} \overset{\;k_{s}}{\to }\\ \underset{\;k_{s}}{\leftarrow } \end{array}\;R_{j}+D_{i}$$

Trans-Estérification:

(4) $$R_{j}+R_{k}\begin{array}{c} \overset{k_{te}}{\to }\\ \underset{k_{te}}{\leftarrow } \end{array}R_{j+k-i}+R_{i}$$

(5) $$R_{j}+D_{k}\begin{array}{c} \overset{k_{te}}{\to }\\ \underset{k_{te}}{\leftarrow } \end{array}R_{j+k-i}+D_{i}$$

(6) $$R_{j}+G_{k}\begin{array}{c} \overset{k_{te}}{\to }\\ \underset{k_{te}}{\leftarrow } \end{array}\;R_{j+k-i}+G_{i}$$

Non-radical Radom Chain scission:

(7) $$R_{j}\overset{k_{de}}{\to }R_{i-k}+G_{k}$$

(8) $$D_{i}\overset{k_{de}}{\to }D_{j-k}+G_{k}$$

(9) $$G_{i}\overset{k_{de}}{\to }G_{i-k}+G_{k}$$

where *k*_a1_, *k*_a2_: Activation rate coefficients; *k*_p_, *k*_d_: Propagation rate coefficients; *k*_s_: Chain-transfer rate coefficient; *k*_te_: Trans-esterification rate coefficient; *k*_de_: Random chain scission reaction rate coefficient; C: catalyst, Sn(Oct)_2_; A: octanoic acid (OctOH) produced by catalyst; R*_i_*: Active polymer chain with length *i*; D*_i_*: Dormant polymer chain with length *i*; G*_i_*: terminated polymer chain with length *i*, M: monomer.

In the reaction mechanism, Equation (1) represents the activation of catalyst (C), Reaction 2 represents the propagation stage of monomer and Reaction 3 signifies the chain termination part of the reaction. The Equations (4)–(6) are side reactions known as intermolecular trans-esterification. Similarly, Equations (7)–(9) are side reactions called non-radical random chain scission which mostly occur at high reaction temperatures. [A], in Equation (1), is treated as a concentration of the octanoic acid which was produced as a bi-product/acid impurity as a result of the reaction of the catalyst and alcohol initiator. The role of AE considered as “natural” acid impurity in the fresh monomer bulk as a result of the impact of [A] is crucial and consequently must be considered.

The reaction kinetic parameters involved in the reaction mechanism such as reaction rate constants (*k* values) *k*_p_, *k*_d_ and monomer equilibrium concentration (*M*e = *K*_p_/*K*_d_), *etc.* were calculated by the fitting of experimental data using a mathematical formulation. The value of *M*e was calculated by considering the experimental conversion (*X*) value with the formula = *M*_0_ × (1−*X*) and *X* = (1−*M*e/*M*o) × (1−e^−*K*pt^). The estimation of *M*e was calculated through curve fitting of the equation in the form of (Y = m × *X* + C or Y = C + A × e^B*X*^). In general, *M*e was formulated as *M*e = slope × *T* + intersect. Propagation rate constant (*k*_p_) was calculated using the equation of the variation of monomer concentration according to the equation $\frac{d\left[M\right]}{dt}≅-kp\;\times R_{T}\times (\left[M\right]-\left[Me\right]),$ where Me is the monomer equilibrium concentration, *M*_0_ is the initial monomer concentration and $R_{T}$ is the initial concentration of the active catalyst. Other parameters including initial rate constants *k*_a1_, *k*_a2_, *k*_te_ and *k*_de_ were taken directly from the literature [29]. This reference covers mostly the details of research work related to mathematical modelling of the ROP process of lactide over the last decade

### 3.2. Modelling of ROP Reaction Mechanism in Reactive Extrusion

Ludovic^®^ is one of the most widely used software packages for the simulation of extrusion processes [30,32,33]. At present, commercial versions of this software are not available with an option to simulate reactive extrusion, *i.e.*, when a reaction happens during the extrusion process (within the extruder barrel). In order to adapt the software for accurate simulation of ROP of PLA, it was necessary to introduce the effect of the reaction happening within the extruder barrel to the properties of the system, *i.e.*, variation of the concentrations of initiator, monomer, polymer and their varying percentages as the reaction progresses. 

Ludovic^®^ software today has the capacity to simulate the extrusion process (considering all thermal and mechanical degradation processes, *etc.*). It is available commercially and is used by industries to fine-tune their throughputs. Ludovic^®^ software works by breaking down the extrusion process (along the barrel) into small subsections (nodes) and using the (input) isothermal curves (molecular weight *vs.* time, viscosity *vs.* time, shear rate *vs.* time, *etc.*) to predict the outcome of the process of extrusion over each of the subsections. These results during each subsection are integrated over the extrusion barrel. As the reactive extrusion processes (such as that of PLA) take hold in the industry, Ludovic^®^ software will have to be updated to take into account the effect of the continuous reactive extrusion. This means the software will have to be upgraded to take into account the effect of the chemical reaction (batch process modelling) as well as the effect of the extrusion process. Hence, isothermal curves (obtained from the mathematical modelling of the batch process) depicting the ring opening polymerization of PLA process were developed (as in ref [29]) and the Ludovic^®^ software was upgraded to accept these as inputs. Unlike traditional extrusion, the molecular weight undergoes drastic changes (monomer to polymer) during this process and, hence, such attempts at inputting a reactive extrusion model into the Ludovic^®^ software will have to be validated through experiments.

**Figure 2:**
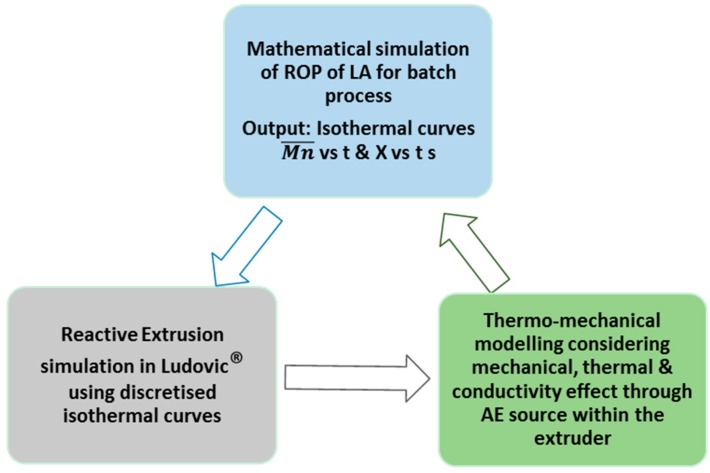
Input and output for continuous extrusion simulation.

To implement this, isothermal curves for the variation of $\bar{M_{n}}$ and *X* with time were generated with the mathematical model based on a batch process reaction mechanism. Some of the properties of the system can be calculated using the degree of polymerization, given by the conversion and average molecular weight, as they represent the percentage of monomer and length of polymer chain at a particular time of reaction. Thus, Ludovic^®^ uses these isothermal curves as initial input. Then, the thermodynamic behavior of the polymerization reaction at several stages can be calculated. The variation provides the initial start-up and output at various stages, as the reaction proceeds through the extruder. A flowchart describing the development of the whole simulation process is presented in Figure 2.

#### 3.2.1. Mechanism of Extruder Reaction through Ludovic^®^

**Figure 3:**
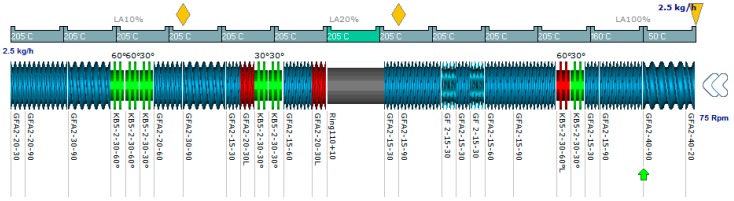
Screw profile with AE source device.

Simulation through Ludovic^®^ detailed the thermo-mechanical behavior of twin screw extruders [34]. It deals with the parameters which directly affect the reaction output such as residence time of mixed material, rate constants, viscosity and thermo-mechanical flow. Stationary thermo-mechanical flow is computed on the basis of:

- Geometrical discretization of the channel zone–C chamber of the screw and material flow of the mixture, as shown in Figure 3
- Changes in pressure and temperature are computed for discrete volumes. Temperature and pressure both updated from the exit to the upstream part of the screw
- The computational calculation was performed in a regular iterative way

To determine the extent of the temperature effect, Ludovic^®^ provides the temperature at each C-chamber zone. This temperature is the result of mechanical, conduction and alternative energy applied within the extruder. The geometry of the extruder, rotation speed of the screw, throughput and melt influence are factors that affect the mechanical energy from shearing; and this impacts the thermal response of the material. 

#### 3.2.2. Alternative Energy Implementation in Ludovic^®^ Software

To the best of the authors’ knowledge, application of AE source within the Ludovic simulator has not been reported in the literature. The AE source can be implemented as a source of heat or thermal impact on the reaction mechanism. Due to its unique application, the effect of the AE source on reaction is different to those that occur during conventional heating (Heat Furnace). The AE source device was attached to the barrel (Grey Zone) in Ludovic^®^ Figure 3. The two specific requirements are

1. Hollow cylinder zone (Non-metal screw, kneading, *etc.*)
2. Fully filled zone

To ensure the application of the AE source is effective, the fully filled zone is to be designed under the alternative energy input section. Figure 3 [34] shows an example of the screw configuration setup with an AE source barrel (chamber in grey color).

#### 3.2.3. Thermo-Mechanical Modelling of Ludovic

Two crucial physical parameters—conductivity and mechanical dissipation—were considered for the thermal balance equation. This equation includes a third term for the source power of the AE, in the AE source barrel component in the model. The updated heat equation is:

(10) $$\rho \times C_{P}\times dT=W_{\mathrm{dissipation}}+W_{\mathrm{conduction}}+W_{AE}$$

**Figure 4:**
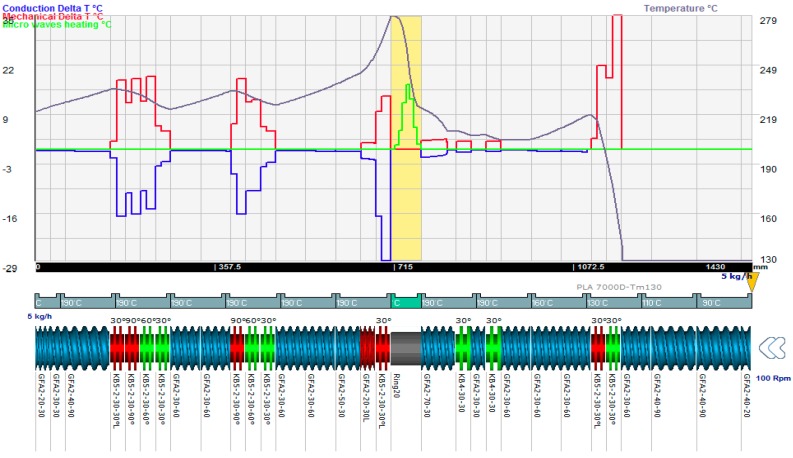
Temperature variation due to conduction (**purple**), due to mechanical effect (**red**) and micro-wave (AE) (100 W) (**green**) effect and result on final temperature (**grey**).

This equation is then computed in each element and Ludovic^®^ provides the variation of temperature due to the AE source (Figure 4) with the associated effects of mechanics and conduction. Finally, these three effects are combined in order to finalize the global evolution of temperature within the twin screw extruder [34]. 

Figure 4 shows the contribution of three different effects of the variation of temperature. The feeding zone is at the right corner and the mixture flows from the right to left side. In fact, in the AE source zone, no metallic material (barrel) is mounted. Due to the use of the non-metallic ring (no metal screw), the shear effect is negligible and only the AE source acts as the source of heating. The heating effect of the AE source is not higher than the dissipation energy (when comparing the magnitude of the green curve to the red curve), but, due to the high concentration of energy being contained a small space, and with no barrel cooling being applied, the increase in temperature is much more significant.

For the implementation of alternative energy in the reaction process, a microwave source or ultrasound Sonification seems to impact the polymerization reaction system [21,23,24,26,28]. The AE power impact is taken into account as a third term (in addition to conduction and dissipation power) in the thermal balance Equation (10).

## 4. Results & Discussion

### 4.1. Experimental Results

Data of average residence time (RT), temperature (*T*), the number average molecular weight ($\bar{M_{n}}$), average molecular weight ($\bar{M_{w}}$) and conversion (*X*) from the reactive extrusion experiments using ultrasounds for initial mixing are provided in Table 2. It was observed that an increase in temperature results in an increase in $\bar{M_{n}}$ , $\bar{M_{w}}$ and *X* for the same initial conditions. Also, $\bar{M_{n}}\;$, $\bar{M_{w}},$ and *X* increases when the Ultrasound (AE) source was applied. In addition, for the experiment at 190 °C, the value of conversion was found to be higher when ultrasound was applied (80%) than in the case without ultrasound (58%). Similar trends of an increase in conversion (*X*) were also observed at a higher temperature. Therefore, the initial step of mixing monomer and catalyst with ultrasound enhances the performance of the reactive extrusion reaction.

#### Impact of AE Source on Extrusion Experiment

The ROP process for PLA production using ultrasound as one of the alternative energy sources in the twin screw extruder depends on several factors such as temperature, throughput, screw rotation speed, *etc.* For the polymerization of lactide, mixing is a crucial factor. The design of several mixing chambers within the extruder and the speed of the twin screw influence the propagation rate of PLA synthesis [35]. The merits for using ultrasound as an AE source, apart from controlling the reaction process, are as follows:

- accelerates the polymerization of the lactide
- enables polymerization at the low rotation speed observed during trial
- (the heating effect which is a side-effect of the sonication treatments (Ultrasounds)) seems to influence the polymerization positively

From this set of experiments in Table 2, the most efficient set of conditions for the experiment is 205 °C, 600 rpm and using Ultrasound (AE). 

The results shown in Table 2 show a positive impact of the AE source on the reaction mechanism and process output. At a high reaction time and temperature, in the presence of the AE source, the values of $\bar{M_{n}}$ and $\bar{M_{w}}$ are high. At a higher temperature, a similar effect is also reported in Table 3, with data calculated through mathematical simulation. In order to perform the simulation considering the reactive extruder condition, the simulation data obtained by the batch process will be used as an input for Ludovic^®^ software which calculates the final result of the reactive extrusion process and will then be compared with the experimental result to facilitate reaction parameter details for the production of PLA at commercial levels.

### 4.2. Results of Batch Process Simulation

To conduct the mathematical simulation of ROP of PLA with the reaction mechanism suggested, initial reaction input details were adopted from the experiments on reactive extrusion. Details are outlined in Table 4.

Based on experimental parameters and results, the mathematical simulation was performed for the reaction times and temperature shown in Table 3. The results are presented in Table 3, including temperature (*T*), number average molecular weight ($\bar{M_{n}}$), average molecular weight ($\bar{M_{w}}$) and conversion (*X*). As a consequence of *A*_0_ being a "natural" acid impurity in the fresh monomer bulk, the impact of *A*_0_ is crucial and needs to be considered.

An increase in temperature supports the propagation stage of the reaction. In general, it favors higher propagation to support the growth of polymer chain. Growth in the chain can be feasible up to a certain range of temperatures. At very higher temperatures beyond 210 °C, side reactions (chain scission and trans-esterification) dominate and reduce the $\bar{M_{n}}\;$which is not suitable for polymerization. The result mentioned in Table 3 shows the increase of $\bar{M_{n}}$ and *X* as temperature increases. This is manifested by an increase in $\bar{M_{n}},\bar{\;M_{w}},$ and *X*. The calculated values of $\bar{M_{n}}$ and $\bar{M_{w}}\;$through batch process simulation reveal higher values than those obtained through the reactive extrusion experiments, although the trends are similar. On the other hand, the rates of conversion obtained in the simulation processes are close to the total conversion (around 95%) at a very high temperature (300 °C), however, these results are based on the modeling of a batch process reaction set-up, in which perfect mixing conditions were assumed. 

#### Isothermal Inputs for Large-Scale Extrusion in Ludovic^®^

**Figure 5:**
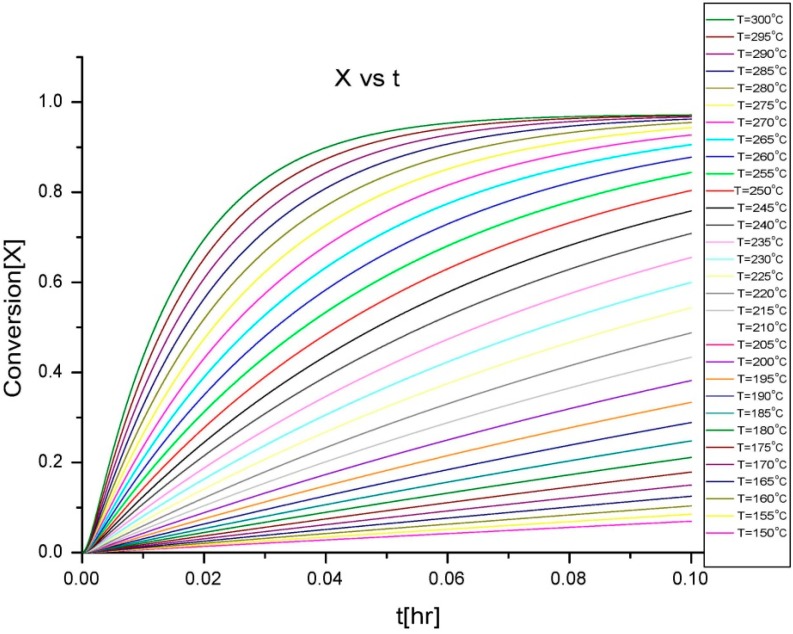
Isothermal curves for conversion (*X*) *vs. t*.

**Figure 6:**
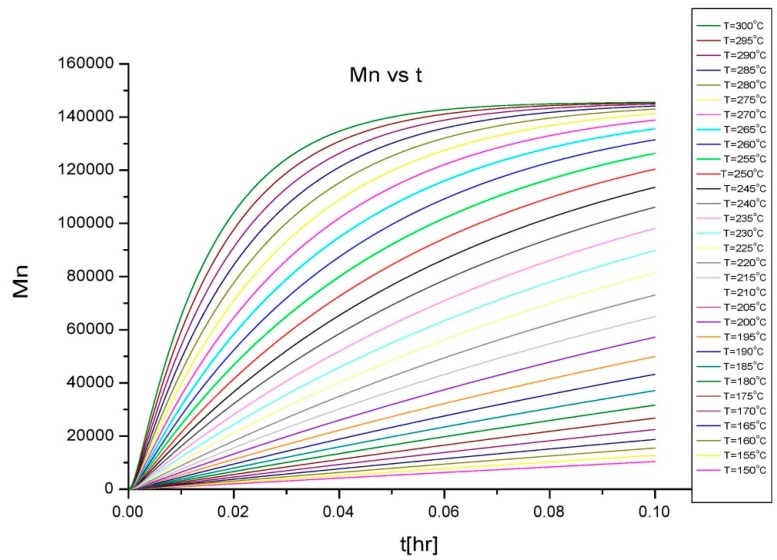
Isothermal curves for average molecular weight ($\bar{M_{n}}$) *vs.* *t*.

The isothermal curves (obtained from the batch process simulation (Figure 5 and Figure 6), conversion (*X*) and number average molecular weight $\bar{M_{n}}$) with time, for the total reaction time of 6 min and temperature range of (150–300) °C were developed to facilitate input for Ludovic^®^. The duration of the input data was based on the residence time reported by experiments on reactive extrusion of the PLA. Details of the input data are shown in Figure 5 and Figure 6. 

### 4.3. Results of Reactive Extrusion Simulation (Ludovic^®^)

**Figure 7:**
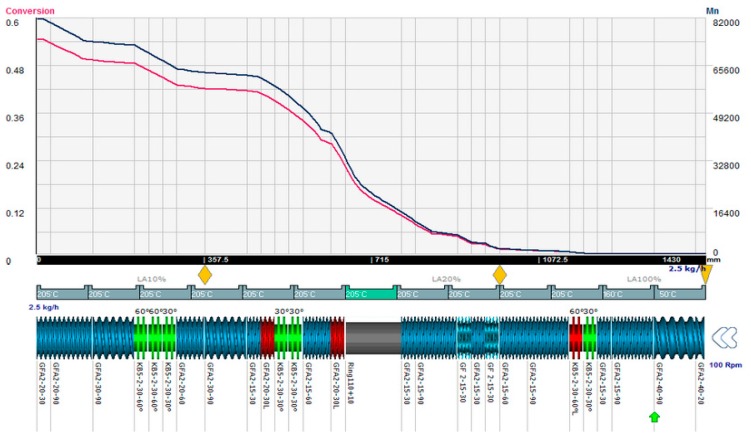
$\bar{M_{n}}$ (**purple line**) *vs.* *T* & *X* (**red line**) *vs. T* obtained with Ludovic^®^ for *T* (50–220) °C, AE = 250 W, 600 rpm.

Figure 7 represents the variation of conversion (*X*) and number average molecular weight ($\bar{M_{n}}$) along the length of the screw (which in a continuous process can be correlated with time for the steady-state) obtained with Ludovic^®^, taking into account the effect of the polymerization. These results correspond to the simulation of the experiment with these initial conditions: temperature 50–220 °C, AE source (250–600) W, screw speed 300–600 rpm. Similar curves have been generated for each reactive extrusion experiment.

### 4.4. Comparison of Extrusion Experiment and Ludovic^®^ Simulation Results

#### Validation of Reactive Extrusion Output from Ludovic^®^

(a) RTD comparison with throughput and barrel temperature

**Figure 8:**
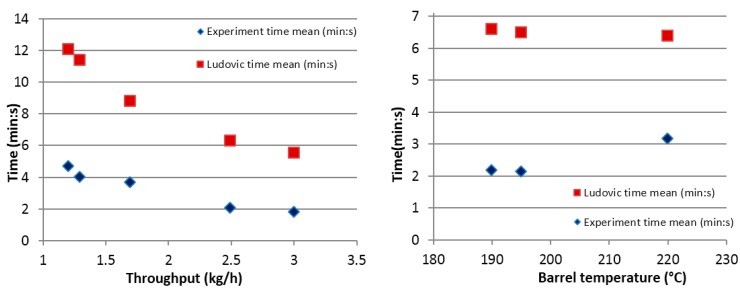
RTD Comparison for Ludovic simulated (**red** dots) and experimental (**blue** dots) results for throughput and barrel temperature.

The initial trials were performed comparing local residence time (RT) for the extrusion reaction experiments and Ludovic^®^ simulations. The effects of throughput and barrel temperature were considered in these trials. Significant differences were found between the experimental and simulated results, as Figure 8 Trial-1 shows. With the implementation of the same initial conditions in the simulation as used in experiments, the result was expected to be similar However, this proved not to be the case.

Trial-1 measurement was based on the assumption that the reaction starts at the beginning of the extruder.

**Figure 9:**
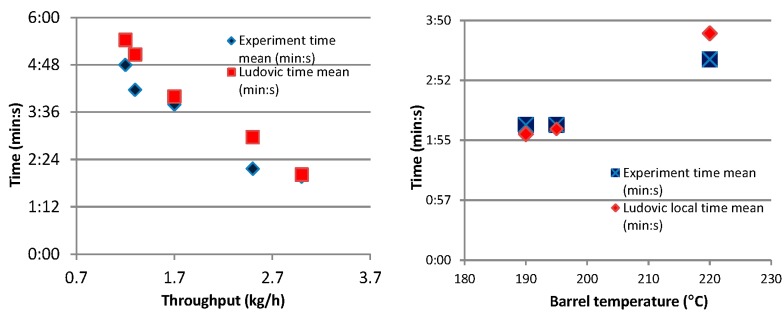
Trial-2 RTD Comparison considering reaction at the middle stage of the extruder.

**Figure 10:**
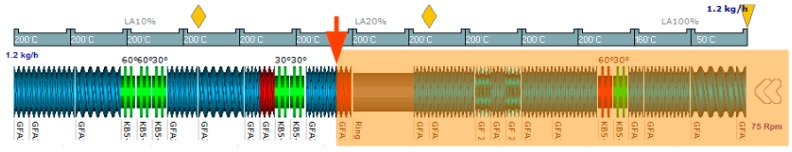
RTD Comparison for Ludovic simulated (**red** dots) and experimental (**blue** dots) results for throughput and barrel temperature.

To determine why the values (RT values) differ, even though the reaction conditions were the same, further consideration was given to the reaction mechanism, and the measurement process was repeated. During reactive extrusion, in the first few seconds, the mixture (catalyst, monomer, initiator, *etc.*) has low viscosity, with flows through the barrel not sheared or extruded. At this stage, only the effect of temperature influences the mixture and the progress of the reaction. Thus, at this point (in the barrel), the reaction progresses like a batch process until the viscosity increases. This is due to the continuous conversion of the monomer into a higher viscosity polymer. After this point, the mixture undergoes further reactions along with shearing forces and extrusion. This behavior (reaction starting in the barrel and not at the feeding stage) was observed during the reactive extrusion experimental trials and, hence, was implemented as an assumption in Ludovic software. Based on this assumption, the barrel length available for extrusion simulation was adjusted to half of the barrel length. This assumption leads to simulation outputs that are comparable to the experimental outputs (Figure 9 and Figure 10).

With regards to the throughput which varied in the range 1–3.5 kg/h, Ludovic^®^ provides results and trends that are similar to the ones from the experiments (Figure 9). Experimental data and simulated results show a similar trend of decrease in RTD while the throughput increases. For the barrel temperature sensitivity (varied in the range 180–220 °C), Ludovic^®^ simulation demonstrates a lower effect in comparison to the experimental one.

It has to be stressed that the impact of the viscosity of the mixture was taken into consideration at three stages of the extruder barrel: first, at the beginning with the higher amount of lactide, then in the middle with a lower amount of lactide monomer, and at the very end with polymerized lactide and the remaining lactide in the reaction. Theoretically, Ludovic^®^ software can calculate the MW within reactive extrusion and then activate the coupling mode in order to link the MW to the viscosity. Implementation of this co-relation will be the subject of a future study. 

(b) Average Molecular weight comparison at various temperatures

Simulation and experimental results for the average molecular weight ($\bar{M_{n}}$) at various temperatures show that, at low temperatures, the $\bar{M_{n}}$ value reported through Ludovic^®^ is higher than that for the experiment. This is related to the higher residence time (RT) reported through Ludovic^®^ than the experiment (Figure 8). If RT is high, it means the material mixture remains for a long time in the mix chambers which facilitates the proper polymerization process and produces the high output. Proper mixing of material in the extruder chamber is one of the key features for optimum polymerization. As temperature increases, due to the effect of side reactions, $\bar{M_{n}}$ goes down. At the same time, exact calculation and proper control of $\bar{M_{n}}$ at high temperatures in the extrusion experimental set-up is not easy.

Applying the hypothesis suggested in the previous section (that only part of the barrel acts as an extruder due to mixing conditions), simulations with Ludovic^®^ were repeated. In this case, the values of $\bar{M_{n}}$ are as expected by Ludovic^®^. The results are shown in Table 5. 

## 5. Conclusions

In this work, reactive extrusion experiments with ultrasound as an AE source were investigated at ICT (Germany). Reactive extrusion was carried out with and without the application of ultrasound at different temperatures, and it was found that the use of ultrasound increases the average number molecular weight ($M_{n})$ of the product in all cases, with an increase of 46%, 96% and 119.7% for 190, 200 and 205 °C, respectively. This demonstrates that the application of an AE source aids the polymerization of lactide and can be used to design a lower energy consuming (and more eco-friendly) process. Also, a simulation model that allows the optimization of reactive extrusion was developed by implementing the mathematical model for ROP of lactide in Ludovic^®^ software. This step is critical from an industrial perspective as it will help in the up-scaling of production of PLA and future commercialization. Ludovic^®^ software provides a great deal of information regarding mixing of material and the residence time distribution of the mixture in the extruder chambers. Up to this point in time, according to the literature, it has not been possible to account for reactions inside the extruder barrel. In this work, isothermal curves of conversion (*X*) and number average molecular weight ($\bar{M_{n}}$) variation with time (*t*) were implemented in Ludovic^®^ software. The isothermal curves were obtained by applying the mathematical model developed and validated in a previous work by Dubey *et al.* [29]. 

Upgraded Ludovic^®^ software was then used to simulate the reactive extrusion of PLA assisted by the AE source. This was performed by SCC, and the filling ratio profile and residence time of mixture material within the extruder were calculated. Initially, significant differences were observed between experimental and simulated results. The predicted values were three times higher than the experimental results in some cases. This led to the assumption that extrusion occurs in the latter part of the barrel, and not from the beginning. However, further studies are required to confirm this theory, such as assessing the actual values of viscosity inside the barrel with the resulting incorporation of this effect into the calculations. 

Further investigations will involve the development of an organometallic catalyst to replace Sn(Oct)_2_ and the adaptation of the mathematical model to the new catalyst system. In addition, consideration of other AE sources such as microwave and LASER will be investigated. Data obtained through Ludovic^®^ will help to set-up and upscale PLA production facilities. 

## Figures and Tables

**Table 1:** Extrusion reaction parameters.

Symbol	Parameter	Value (Unit)
*M*_0_	Initial concentration of monomer	8.3 mol/L
*C*_0_	Initial concentration of catalyst	0.008 mol/L
*D*_0_	Initial concentration of co-catalyst	0.008 mol/L
*T*_0_	Initial temperature	50 °C
*M*	Monomer mass flow	1.2–2.5 m/kg
RPM	Rotational speed	75 or 600 rpm

**Table 2:** Reactive extrusion experimental data.

RT (min)	*T* (°C)	Ultrasounds	$\bar{M_{n}}$ (g/mol)	$\bar{M_{w}}$ (g/mol)	RPM	*X* (%)
2.30	190	Yes	12,396	15,500	75	80
		No	7,720	10,600	75	58
6.10	200	Yes	14,800	20,200	75	92
		No	13,500	17,600	75	85
7.3	205	Yes	30,100	50,000	600	94
		No	13,700	18,400	600	86

**Table 3:** Mathematical simulation results for ROP of PLA in batch process.

*t* (min)	*T* (°C)	$\bar{M_{n}}\;$ (g/mol)	$\bar{M_{w}}$ (g/mol)	*X* (%)
2.30	190	8,500	11,645	50
6.10	200	31,100	40,430	74
7.3	205	35,000	87,500	82

**Table 4:** Initial reaction parameters for simulation.

Symbol	Parameter	Value (Unit)
*M*_0_	Initial concentration of monomer	8.326 mol/L
*C*_0_	Initial concentration of catalyst	0.008 mol/L
*D*_0_	Initial concentration of co-catalyst	0.008 mol/L
*T*_0_	Initial temperature	50 °C
*A*_0_	Octanoic acid (OctOH)	0.24 mol/L
*M*e	Monomer equilibrium concentration	0.225 mol/L

**Table 5:** Comparison between simulation results (“Ludovic^®^”) and experimental results (“Expt”).

S.No	Temp (°C)	$\bar{M_{n}}$ (Expt.)g/mol	$\bar{M_{n}}$ (Ludovic^®^)g/mol
1	190	7,700	10,000
2	200	14,500	16,000
3	205	31,000	25,000
